# Analysis of Heart Rate Variability Before and During Tilt Test in Patients with Cardioinhibitory Vasovagal Syncope

**DOI:** 10.5935/abc.20160177

**Published:** 2016-12

**Authors:** Cláudia Madeira Miranda, Rose Mary Ferreira Lisboa da Silva

**Affiliations:** 1 Serviço de Cardiologia do Hospital Madre Teresa; Belo Horizonte, MG - Brazil; 2 Universidade Federal de Minas Gerais, Belo Horizonte, MG - Brazil

**Keywords:** Heart Rate, Syncope, Vasovagal / physiotaphology, Tilt-Table Test, Electrocardiography, Ambulatory

## Abstract

**Background:**

Cardioinhibitory vasovagal response is uncommon during the tilt test (TT). Heart rate variability (HRV) by use of spectral analysis can distinguish patients with that response.

**Objective:**

To compare the HRV in patients with cardioinhibitory vasovagal syncope (case group - G1) with that in patients without syncope and with negative response to TT (control group - G2).

**Methods:**

64 patients were evaluated (mean age, 36.2 years; 35 men) and submitted to TT at 70 degrees, under digital Holter monitoring. The groups were paired for age and sex (G1, 40 patients; G2, 24).

**Results:**

In G1, 21 patients had a type 2A response and 19 had type 2B, with mean TT duration of 20.4 minutes. There was a greater low frequency (LF) component (11,6 versus 4,5 ms^2^, p=0.001) and a lower low/high frequency ratio in the supine position (3,9 versus 4,5 ms^2^, p=0.008) in G1, with no difference during TT between the groups. Applying the receiver operating characteristic curve for cardioinhibitory response, the area under the curve was 0.74 for the LF component in the supine position (p = 0.001). The following were observed for the cutoff point of 0.35 ms^(2)^ for the LF component: sensitivity, 97.4%; specificity, 83.3%; positive predictive value, 85.3%; negative predictive value, 96.9%; and positive likelihood ratio, 5.8.

**Conclusion:**

HRV in the supine position allowed identifying patients with syncope and cardioinhibitory response with a high negative predictive value and likelihood ratio of 5.8.

## Introduction

Syncope is the transient and abrupt loss of consciousness secondary to cerebral hypoperfusion, of short duration and spontaneous recovery.^1^ In the Framingham study, its incidence was 6.2/1000 people-year, the vasovagal being the most frequent type (21.2%).^2^ Although not completely clarified, the pathophysiology of vasovagal syncope is characterized by a reflex activation that triggers a rapid increase in heart rate (HR) and a reduction in vascular tone, resulting in arterial hypotension and/or bradyarrhythmia.^1,3^

Tilt test (TT) is used to diagnose vasovagal syncope, being safe, of low cost and of good reproducibility.^1,3-5^ The final response to TT is the reflex induction of arterial hypotension and/or bradycardia, associated with syncope or presyncope, which is classified as vasodepressor, mixed or cardioinhibitory. The latter manifests with or without asystole (2B, with asystole for more than 3s; or 2A, HR < 40 bpm for more than 10s; respectively).^6^ The incidence of that response varies from 1 to 4.4% of the positive tests, reaching 21% in protocols sensitized with nitroglycerin and 13% in protocols sensitized with isoproterenol, being more frequent in young individuals.^7-9^

Previous studies have shown changes in HR variability (HRV) during TT with gradual inclination in healthy patients, providing a non-invasive quantitative analysis of the vagal sympathetic balance, via its components of low frequency (LF), high frequency (HF) and LF/HF ratio.^10,11^ There are only four studies^12-15^ including spectral analysis of adult patients with cardioinhibitory response and distinct behavior, evidencing an increase in the LF component at rest, a greater reduction in the HF component after TT, or an increase in the LF/HF ratio before the syncopal event during TT, using only univariate analysis and no receiver operating characteristic curve for the analysis of the HRV predictive value regarding that specific response. Thus, the present study aimed at assessing HRV by using spectral analysis before and during TT in patients with vasovagal syncope and cardioinhibitory response, and at comparing it with the HRV of patients with negative response to TT and no history of syncope, assessing its predictive value.

## Methods

This is a case-control, observational, prospective study, whose population sample comprised 64 patients consecutively selected to undergo TT at the Department of Graphic Methods of the Hospital Madre Teresa, from January 2013 to February 2014, from a total of 435 patients. They were divided into two groups: case group, 40 patients with history of syncope and cardioinhibitory response on TT; and control group, 24 patients with other symptoms not related to loss of consciousness, such as dizziness or fall, and neither syncope nor presyncope, with a negative TT (no symptoms, neither vasovagal response nor dysautonomia). Both groups had sinus rhythm and were paired for sex and age. Patients aged at least 14 years (the age group cared for at the hospital), of both sexes and able to undergo TT were consecutively included. The following were excluded: pregnant women, patients refusing to participate in the study, patients with coexisting conditions that could affect the HRV analysis, such as atrial fibrillation, pacemaker rhythm and use of antiarrhythmic drugs, and patients undergoing heart transplantation.

The population size was calculated as 52 patients, and such calculation was based on the 1:1 ratio between the two groups, standard deviation of the spectral analysis components of 200 ms^2^, minimum difference to be detected of 100 ms^2^, test power of 90%, significance level of 5%, and one-tailed test. In addition, the number of participants included in similar studies was considered.

This study project was approved by the Ethics Committee in Research, and the participants provided written informed consent.

All TTs were performed in the morning period, using a tilt table with an angle ranging from -20 degrees (*Trendelenburg* position) to 70 degrees (upright), support for the patient's feet, in a quiet room under mild and constant temperature. The fasting patients were allowed to rest in the supine position for 10 minutes, and then tilted (70 degree) during the first 20-minute step. When there was no event (syncope or presyncope), the drug-sensitization step was initiated with the sublingual administration of 1.25 mg of isosorbide for up to 15 minutes. In the presence of events, such as cardioinhibitory response with symptoms, the test was considered positive. Simultaneously, continuous electrocardiographic monitoring was performed, as well as intermittent recording of blood pressure every 3 minutes by use of the Hewlett Packard Omnicare 24C monitor. Continuous electrocardiographic recording was performed by use of the Holter system with a digital recorder (DMS 300-8) of three channels (V1, modified V5 and D3) to analyze HRV in the supine position for 10 minutes and in the tilt position. Recording was performed on the fifth minute (during the last minute and for a total of 5 minutes) in the supine and tilt positions for all patients. In addition, recording was performed on the fifth minute at the end of inclination, in the control group, and on the fifth minute after an event, in the case group in the supine position.

The software Holter DMS, version 76, was used for the HRV spectral analysis, assessing the LF, HF and LF/HF ratio components via the Fourier mathematical model, after processing the data obtained, with correction of extrasystoles and artifacts. The results of that analysis were expressed in absolute units (ms^2^).

The SPSS (Statistical Package for the Social Sciences) software, version 14.0, was used for data analysis. The results were expressed as numbers and proportions for categorical variables, and as measures of central trend and dispersion for continuous variables. Mann-Whitney and chi-square or Fisher tests, when appropriate, were used to compare the differences between continuous and categorical variables (nominal or ordinal), respectively. Wilcoxon test was used to compare HRV values between the periods of supine position, during inclination and after the event or end of inclination. Logarithmic transformation of HRV values was performed. Receiver operating characteristic curve was used to assess sensitivity and specificity of HR spectral analysis in the supine position, considering the positive response to the test. The significance level adopted was 5%.

## Results

### General characteristics of the case series

The patients' mean age was 36.2 ± 17.9 years (range, 14 - 77), 35 (54.7%) being males. Regarding the case group, the median time of symptom presence was 20 months. The mean time since the last syncope episode was 60.1 months. The mean number of syncope episodes was 4.17 ± 2.6 (range, 1 - 12), and the Calgary score ranged from -8 to +4 points (mean, -0.9). The hemodynamic variables of the entire case series are shown in Table 1.

**Table 1:** Hemodynamic variables of 64 patients

Variables	Mean	Standard deviation	Minimum value	Maximum value
supine SBP (mmHg)	119.2	15.2	95	166
supine DBP (mmHg)	61.5	10.4	42	94
supine HR (bpm)	63.4	9.2	48	89

SBP: systolic blood pressure; DBP: diastolic blood pressure; HR: heart rate.

In the case group, 38 patients (95%) reported prodromes, while 8 patients (20%) reported trauma resulting from the syncope episode. The triggers related to syncope were as follows: body posture type (upright or sitting), 31 patients (77.5%); emotional stress, 8 (20%); and sight of blood, 1 patient. To avoid bias in HRV interpretation, the case and control groups were paired for sex and age: 17 women in the case group (42.5% of the group) and 12 women in the control group (50%), p=0.56. In the case group, mean age was 32.9 ± 14.8 years, and, in the control group, 41.7 ± 21.2, p=0.13.

### Clinical and hemodynamic variables during tilt test

During TT, the control and case groups did not significantly differ regarding the hemodynamic variables (HR and blood pressure) in the supine position and during inclination (Table 2).

**Table 2:** Hemodynamic variables during tilt test (TT)

Variables	Control group (n=24)		Case group (n=40)		p Value
	**Mean**	**Standard deviation**	**Mean**	**Standard deviation**	
supine HR	65,5	10,8	62,1	7,8	0,26
supine SBP	12,3	16,6	117,8	14,2	0,52
supine DBP	63,0	11,3	60,5	9,7	0,46
TT HR	79,3	14,7	80,4	13,0	0,72
TT SBP	122,5	14,7	117,6	15,7	0,15
TT DBP	x00A0;	12,6	66,8	11,4	0,79

SBP: systolic blood pressure; DBP: diastolic blood pressure; HR: heart rate.

**Figure 1:**
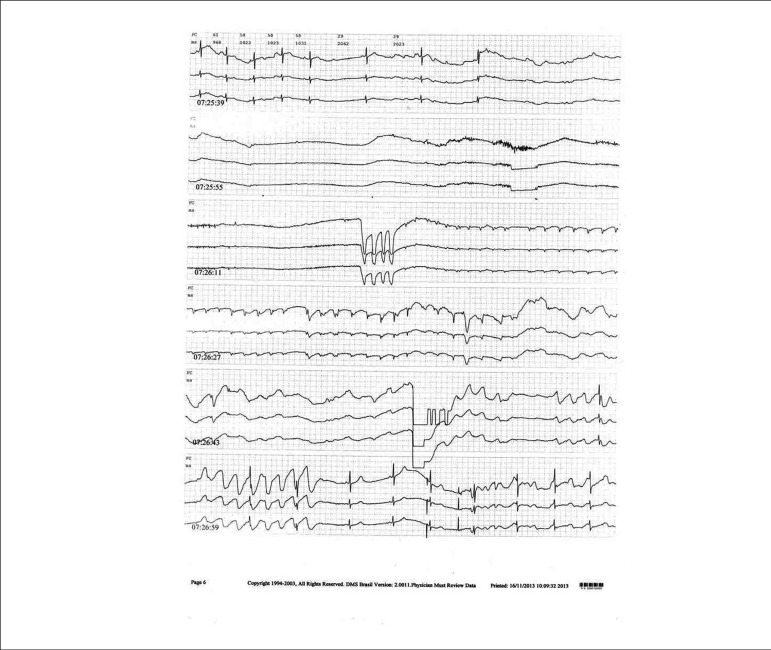
Continuous electrocardiographic recording by use of Holter of a patient with type 2B cardioinhibitory response at 13 minutes of tilt test, with asystole of longest duration (70.2 s).

In the case group, 21 patients (52.5%) had type 2A response, and 19 (47.5%), type 2B response, constituting the subgroups. In the type 2A response subgroup, mean age was 35.9 ± 14.5 years, and, in the type 2B response subgroup, 29.5 ± 14.9 years (p=0,09). The case group had 17 women (42.5%), with 9 women and 12 men in the type 2A response subgroup, while the type 2B response subgroup had 8 women and 11 men. In the type 2A response subgroup, the mean HR achieved was 28.4 ± 5.2 bpm (range, 20 - 38 bpm). In the type 2B response subgroup, the mean value of pause was 14.2 ± 16.5 seconds (range, 3.4 - 70.2 seconds) (Figure 1), while the median value of pause was 9.7 seconds. The mean time for positive response on TT was 20.4 ±7.8 minutes (range, 5 - 34 minutes). Prodromes were reported by 20 patients (95.2%) in the type 2A response subgroup, and by 18 (94.7%) in the type 2B response subgroup (p=0.73). Trauma due to syncope was reported by 5 patients in each group (p=0.57). The triggers related to syncope were mainly the upright position and the sitting position in both 2A and 2B subgroups, with no significant difference (p=0.75). There was no sex predominance regarding the cardioinhibitory response (23 men versus 17 women, p= 0.60). Sensitization during TT was performed in 26 patients (65%) of the case group: 14 and 12 patients of the 2A and 2B subgroups, respectively (p=0.53).

### Spectral analysis of heart rate variability

Comparing the HRV components during the supine position and on the fifth minute of TT by using Wilcoxon test in the entire case series, the mean values of those components and p values were obtained, being shown in Table 3. There was statistical significance in the HF (p<0.0001) and LF/HF ratio (p<0.0001) components when changing the supine position on the fifth minute after inclination; however, the LF component showed no significance (p=0.19).

**Table 3:** Spectral analysis of heart rate variability (HRV) of patients in the supine position and on the fifth minute of the tilt test (TT)

Variables	Supine position	5th min TT	p Value
LF (ms^2^)	8.9	10.0	0.19
HF (ms^2^)	6.2	2.7	0.000
LF/HF	4.1	7.1	0.000

LF: low frequency component of HRV; HF: high frequency component of HRV; ms^2^: milliseconds squared.

When comparing HRV during only 1 minute (on the fifth minute) between case and control groups, by using Mann-Whitney test, there was a significant difference in the supine position regarding the LF and LF/HF ratio components, but no difference between the groups during inclination. Table 4 shows those data and the comparison between the inclination phase, on the fifth minute, and after the test.

**Table 4:** Comparison of the heart rate variability (HRV) components between the case and control groups

x00A0;	Case group			Control group			p Value		
x00A0;	T0	T1	T3	T0	T1	T3	T0	T1	T3
LF (ms2)	11.6	11.0	8.4	4.5	8.4	4.8	0.001	0.11	0.001
HF (ms2)	7.4	2.8	7.3	4.2	2.6	1.3	0.09	0.27	0.000
LF/HF	3.9	8.1	2.5	4.5	5.4	4.3	0.008	0.23	0.07

LF: low frequency component of HRV; HF: high frequency component of HRV; T0: supine position before tilt test; T1: on the 5th min of tilt test; T3: after tilt test.

**Figure 2:**
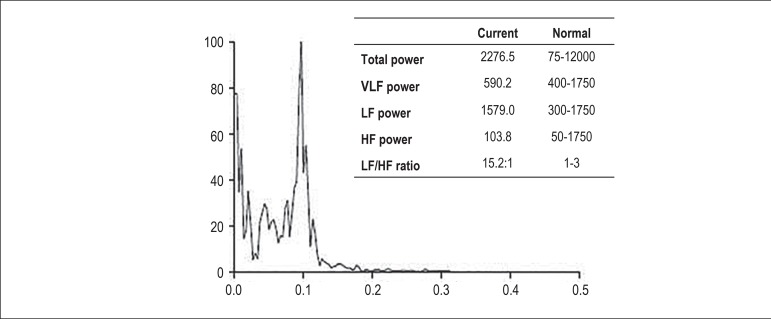
Graph of heart rate spectral analysis of a case group patient during all monitoring. LF: low frequency; HF: high frequency; VLF: very low frequency.

**Figure 3:**
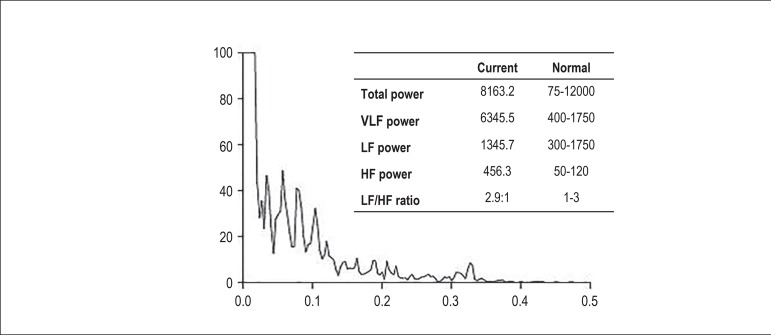
Graph of heart rate spectral analysis of a control group patient during all monitoring. LF: low frequency; HF: high frequency; VLF: very low frequency.

The HRV analysis was also performed during the five minutes accumulated in the supine position between the case and control groups. That analysis showed a significant difference regarding the LF component (963.3 versus 557.0 ms^2^, p=0.004), but no difference regarding the HF (p=0.48) and LF/HF ratio components (p=0.77). During the first five minutes at the beginning of TT (inclination phase), a difference occurred only regarding the LF component (729.0 ms^2^ in the case group, and 532.1 ms^2^ in the control group; p=0.04). During the five minutes after TT, statistically significant difference was observed only for the LF component (543.9 versus 693.0 ms^2^ for the case and control groups, respectively; p=0.22). With logarithmic transformation of those HRV component values, the same p values were obtained. The HR spectral analysis of 1 patient in the case group and of another patient in the control group is plotted in Figures 2 and 3, respectively.

### Analysis of the receiver operating characteristic curve

**Figure 4:**
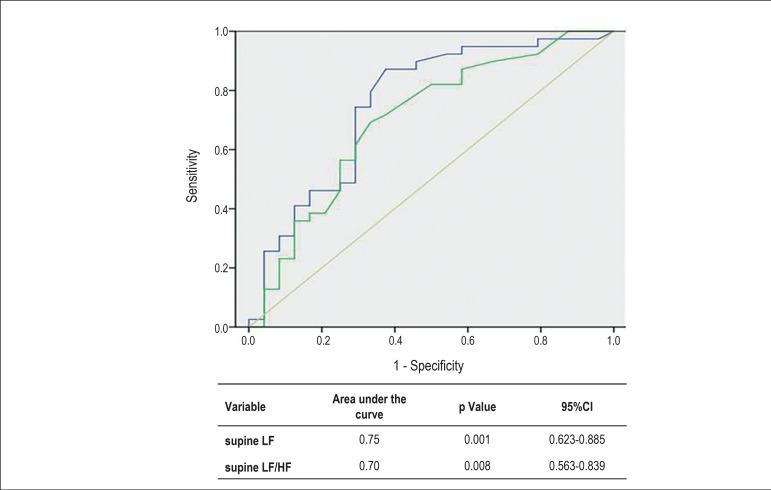
Receiver operating characteristic curve for the LF and LF/HF ratio components in the supine position, with area under the curve, p value and 95% confidence interval (CI) for all case series, considering cardioinhibitory response as the stable variable. LF: blue line; LF/HF: green line. LF: low frequency; HF: high frequency.

Applying the receiver operating characteristic curve for the entire case series, considering cardioinhibitory response as the stable variable, that is the case group, the areas under the curve obtained were 0.74 and 0.70 for the LF and LF/HF ratio components, both in the supine position, respectively, with statistical significance according to the Mann-Whitney test. The curves and data with p values and 95% confidence intervals are shown in Figure 4. The cutoff point of 0.35 ms^2^ for the LF component, considered the best, had sensitivity of 97.4% and specificity of 83.3%. The positive predictive value (PPV) was 85.3% and the negative predictive value (NPV), 96.9%. The positive likelihood ratio was 5.8. For the LF/HF ratio in the supine position, sensitivity was 89.7% and specificity, 66.7%, with PPV of 72.9% and NPV of 86.6%. Considering type 2B response as the stable variable, the receiver operating characteristic curve obtained for the case group showed no statistical significance for any of the HRV components.

## Discussion

The HRV spectral analysis parameters during TT have been shown to help to understand the mechanism of syncope,^10-11^ being useful to identify autonomic changes before and during TT in patients with syncope.^13-15^ The present study showed that the HRV components could predict the cardioinhibitory response before beginning the tilting, and some of them, when compared in the different phases of TT, differed from those in the control group. The LF component in the supine position, with better discriminatory power than the LF/HF ratio, showed sensitivity of 97.4%, with excellent diagnostic screening, correctly detecting patients with history of syncope, and specificity of 83.3%, also detecting those truly negative, who had no history of syncope, enabling diagnostic confirmation. The power of that diagnostic test resulted in a high NPV, due to its higher sensitivity, with a positive likelihood ratio, that is, TT's likelihood of a positive cardioinhibitory response of 5.8.

The HRV components in the frequency domain undergo changes during the tilting phases, being influenced by some factors. Comparing HRV in the supine position and after the tilting postural maneuver, healthy individuals showed an increase in the LF and LF/HF ratio components, and a reduction in the HF component regarding the values at rest in the supine position.^14^ To assess the influence of age, Ruiz et al.^16^ have compared HRV data between patients with positive and negative response on TT, divided into two groups according to age (young, 15 to 35 years of age; and elder, over 60 years), and have shown a significant change in the LF components and an increase in the LF/HF ratio between the supine and tilted positions, less evident among the elderly. However, that study has not assessed the cardioinhibitory response. Regarding sex, a study paired for age,^17^ comparing healthy men and women with a mean age of 50 years, has shown in the female sex a lower LF component (p<0.001), a higher HF component (p<0.001) and a lower LF/HF ratio (p<0.001) as compared to the values obtained in male patients. Another study^18^ on HRV has shown that young women have a lower LF component and LF/HF ratio than young men during the postural maneuver. Barantke et al.,^19^ in a study with healthy volunteers, have reported a higher LF component in men as compared to women, both in the supine and tilted positions, as well as no difference in the HF component between sexes, which could justify the difference between sexes regarding orthostatic tolerance.

Because of the influence of age and sex on HRV, and to prevent interpretation bias, the present study compared the group of patients with history of syncope and all with cardioinhibitory response on TT with the control group, paired for age and sex.

Regarding the ability of the HRV components to predict and differentiate patients who would have syncope during TT, Duplyakov et al.^20^ and Kochiadakis et al.^10^ have demonstrated a reduction in the HF component in patients with positive response on TT, in the time period between the beginning of tilting and immediately after the end of TT, which did not significantly occur in patients with negative response. However, Furlan et al.^21^ have shown that different models of spectral HRV can be detected preceding syncope: one characterized by a progressive increase in cardiac autonomic modulation until the sudden occurrence of bradycardia, and another with gradual inhibition of the sympathetic component and concomitant increase in vagal modulation. In accordance with the results of the present study, Kouakan et al.,^22^ assessing 69 patients submitted to TT and with history of unexplained syncope, have concluded that the LF/HF ratio persisted reduced during the entire period of inclination, being the only variable that discriminated the groups with positive and negative responses on TT (p=0.005), with 89% sensitivity, 89% specificity, 92% PPV and 86% NPV.

This subject has distinct results regarding the behavior of HRV spectral analysis. This can be explained by the case series of those studies including patients with history either suggesting vasovagal syncope or unexplained, with different responses on TT and distinct periods of time to assess HRV, in addition to other variables, such as age and sex.

The response on TT, whether vasodepressor, mixed or cardioinhibitory, could reflect different behaviors of HRV; however, those responses on TT have been reported in studies only as subgroups of positive response on TT. Only the vasodepressor response has been emphasized. Prinz-Zaiss et al.,^23^ comparing a group of patients with syncope of vasodepressor response with the control group, have demonstrated that, during tilting, there was a progressive increase in the LF component and a decrease in the HF component. However, during the presyncope period, in the group with syncope, the LF component decreased. In addition, the HF and HF/LF ratio components did not significantly differ between groups.^24^ However, in that study, no patient with cardioinhibitory response was included.

Considering the HRV behavior in patients with cardioinhibitory response on TT, Guzman et al.,^12^ with a small case series, have shown a higher LF component at rest in the first minute after syncope during TT in patients with cardioinhibitory response (9 patients), as compared to that value of patients with vasodepressor response (7 patients). Kochiadakis et al.,^14^ assessing 24 young individuals (mean age, 28 years) submitted to TT and with cardioinhibitory response in 71% of them (17 patients), have reported a decrease in the HF component after TT, as compared to 31 patients with mean age of 56 years, 68% of whom had vasodepressor response on TT. Another study,^15^ in which only 8 patients had cardioinhibitory response, has reported no difference in HRV at rest between those with different responses on TT; however, there was sympathetic activation during TT in those with cardioinhibitory response. Those studies had a small case series and a small number of patients with cardioinhibitory response as compared to the present study, which compared 40 patients with cardioinhibitory response with those in the control group, paired for sex and age, with a more robust design and avoiding interpretation bias.

Although the vasovagal syncope is attributed to the Bezold-Jarish reflex, with paradoxical bradycardia and hypotension due to sympathetic inhibition and subsequent parasympathetic hyperactivity,^1,25^ its pathophysiology is still controversial. Differently from patients with vasodilation in the presyncope period, 34% of those with vasovagal syncope had a decrease in cardiac output, mainly due to a drop in HR, with no change in total peripheral resistance.^26^ In addition, there is evidence of sympathetic innervation activity persistence in patients with vasovagal syncope.^27^ That could explain the findings of the present study, with an increase in the LF and LF/HF ratio components in the case group before the occurrence of syncope or presyncope on TT.

Regarding the clinical profile of the case series of the present study, 9.2% of the patients submitted to TT had cardioinhibitory response, an incidence in accordance to those in the literature: up to 6.6%, considering only the passive phase,^28^ or up to 21% in sensitized protocols.^7-9^ Trauma secondary to syncope occurred in 20% of the case group with exclusively vasovagal syncope, while in the literature that ranged from 27.5% to 29% in a heterogeneous group (vasovagal and of unexplained origin). Prodromes, which are important in clinical history, occurred in 95% of the case group in the present study, being frequently reported in the literature, mainly among young indivduals.^1,29-31^

Considering that the asystole of the TT is a rare response during spontaneous syncope^32^ and that its investigation has better cost-benefit ratio if initiated during TT than by the monitor of implantable events,^33^ further studies on cardioinhibitory response, especially on type 2B response, are necessary.

### Limitations

The results of the present study should not be extrapolated to different and less selected groups (with other types of response on TT) of patients with vasovagal syncope. The acquisition of continuous hemodynamic parameters of blood pressure was limited, because digital pletismography was not used. However, this did not influence the HRV results. The number of patients with cardioinhibitory response with and without asystole was small for appropriate comparison between both subgroups.

## Conclusions

The spectral analysis in supine position, before TT, by use of the LF and LF/HF ratio components, allowed the identification of patients with history of syncope who had cardioinhibitory response, and the comparison to patients with no history of syncope and with negative TT. The case group patients had greater sympathetic activation in the supine position. Thus, the HRV analysis can be used as a non-invasive tool to predict response to TT, with high NPV and likelihood ratio of 5,8.
